# Smoking characteristics and lung functions among university athletes

**DOI:** 10.1038/s41598-020-77248-y

**Published:** 2020-11-18

**Authors:** Narongkorn Saiphoklang, Orapan Poachanukoon, Suchada Soorapan

**Affiliations:** 1 Division of Pulmonary and Critical Care Medicine, Department of Internal Medicine, Faculty of Medicine, Thammasat University, 99/209 Paholyotin Road, Klong Luang, Pathum Thani 12120 Thailand; 2 Department of Pediatrics, Faculty of Medicine, Thammasat University, Klong Luang, Thailand; 3 Faculty of Pharmacy, Thammasat University, Klong Luang, Thailand

**Keywords:** Respiration, Health care, Medical research

## Abstract

Cigarette smoking has negative effects on the respiratory system, particularly pulmonary functions. This study aimed to determine smoking prevalence and characteristics among university athletes. We conducted a cross-sectional study of Thammasat University athletes in Thailand from July to October 2018. Demographic and smoking data were recorded. Exhaled carbon monoxide (CO) levels and lung function data were analysed. A total of 433 subjects (56% men) were included. Mean age was 19.8 ± 1.3 years. Asthma was reported in 5.5%. The prevalence of current cigarette smoking was 23.8%. Tobacco use was 3.0 ± 3.2 cigarettes per day. The Fagerstrom score for nicotine dependence was 0.76 ± 1.47. Compared to non-smokers, smokers were predominately males (70.6% vs 29.4%, *P* < 0.001), had higher exhaled CO levels (3.75 ± 3.08 ppm vs 2.18 ± 0.73 ppm, *P* < 0.001), higher FVC (89.65 ± 17.61% vs 83.22 ± 15.72%, *P* = 0.001), higher FEV_1_ (92.60 ± 15.36% vs 87.77 ± 11.23%, *P* = 0.002), but lower FEV_1_/FVC (78.21 ± 5.38% vs 79.70 ± 5.60%, *P* = 0.015). Moreover, athletes who smoke, were more likely to: drink alcohol, have a family member who smokes, have a friend who smokes or have a university instructor who smokes. In conclusion, smoking prevalence among university athletes was relatively high, although low nicotine addiction level and good lung functions were found. Home and institute environments had important influences on cigarette use in students.

**Trial registration**: TCTR20180917001

## Introduction

Smoking prevalence in undergraduate athletes in the United States was found to be 25% for current smokeless tobacco users and 4% for smokers^1^. As well, the survey of the National College Health Assessment in the United States found varsity athletes and students who were not varsity athletes had similar rates of waterpipe tobacco smoking (27.6% and 29.5%, respectively)^2^. Previous study among professional athletes in Iran found 24.6% of athletes had experienced cigarette smoking and 9% were current smokers^3^. Smoking among Olympic athletes of Finland was reported to be 11.4%^4^.

Fagerstrom test is a smoking-related questionnaire for assessing nicotine dependence. High Fagerstrom scores indicate high nicotine addiction^5^. Exhaled carbon monoxide (CO) test is a simple and rapid measure to distinguish between active smokers and non-smokers. High exhaled CO levels indicates active smoking. In a previous study, exhaled CO cutoff value of 6.5 ppm revealed 90% sensitivity and 83% specificity^6^. Another study showed that exhaled CO cutoff value of 5 ppm reflected 79% sensitivity and 89% specificity^7^.

Cigarette smoking is associated with several conditions such as cardiovascular diseases^8^, chronic obstructive pulmonary disease (COPD)^9^, and cancers^10^. A previous study of smoking in university taekwondo athletes in Korea found that non-smokers had significantly better cardiopulmonary functions during and after exercise than smoking athletes^11^. As well, a previous study of smoking in young athletes found that smokers had lower maximal oxygen uptake, indicating anaerobic limitation, than non-smokers^12^. Moreover, passive smoking had a fourfold increase in incidence of low forced expiration flow rate at 25–75% of forced viral capacity (FEF_25-75_) in high school athletes^13^. As well, a study of passive smoking had influence on many lung functions in young athletes^14^.

Cigarette smoking among university students remains a substantial problem in some countries^15^, however limited data on smoking characteristics and pulmonary functions in university athletes have been published. This study aimed to determine smoking prevalence and characteristics including lung functions among university athletes.

## Methods

### Study design and subjects

A cross-sectional study was conducted at Thammasat University in Thailand from July to October 2018. Student athletes aged 18 years or older were recruited. Exclusion criteria were inability to perform spirometry or exhaled CO tests. Subjects were divided into 5 sport groups; the ball group (e.g., basketball, volleyball, and football), the strength group (e.g., boxing, judo, taekwondo and fencing), the endurance group (e.g., middle distance race and long-distance runners), the static group (e.g., shooting), and the swim group (e.g., swimming and water ballet).

Ethic approval was obtained from the Ethics Committee of Faculty of Medicine, Thammasat University (IRB No. MTU-EC-IM-0–132/61), in compliance with Declaration of Helsinki, The Belmont Report, CIOMS Guidelines and The International Practice (ICH-GCP). All methods were performed in accordance with these guidelines and regulations. All participants provided written informed consent.

### Procedures and outcomes

Subjects’ demographic data, comorbidity, and exercise frequency were recorded. All subjects completed smoking-related questionnaires including baseline characteristics, smoking characteristics, and level of nicotine dependence. Current smoker was defined persons who reported smoking during the past 30 days^16^. Exhaled CO levels were tested as an easy, noninvasive, and objective tool to assess smoking behavior^17^ using exhaled CO test (Micro Smokerlyzer CO monitor, Bedfont Scientific Ltd, Rochester, England). For CO testing, participants were instructed to take a deep breath, hold for 20 s, and then exhale slowly. Exhaled CO level was reported in parts per million (ppm). Spirometry was performed according to American Thoracic Society and European Respiratory Society guidelines^18,19^ using PC spirometer (Vyntus SPIRO, Vyaire Medical, Inc., Mettawa, IL, USA). Forced expiratory volume in one second (FEV_1_), forced vital capacity (FVC), FEV_1_/FVC, and FEF_25-75_ were recorded and reported in liters (L), %predicted, %, or liters per second (L/s). Bronchodilator reversibility test indicating asthma was done using salbutamol inhalation (total dose 400 µg) and repeating spirometry 15 min later according to American Thoracic Society and European Respiratory Society guidelines^19^. This procedure was performed only if asthma was highly suspected because a subject had history of asthma or allergic rhinitis, or FEV_1_/FVC < 90%predicted or FEF_25-75_ < 70%predicted.

The current version of the Fagerstrom test was used for assessment of nicotine dependence^20^. It includes 6 items and is brief; its completion requires a few minutes^5,20^. Scores of 0–2 indicate low dependence, scores 3–4 indicate low to moderate dependence, scores 5–7 indicate moderate dependence, and scores ≥ 8 indicate high dependence^20^.

### Statistical analysis

Categorical data was shown as number (%). Continuous data was shown as mean ± standard deviation. Chi-squared test was used to compare categorical variables between two groups. Pearson correlation was used for correlation analysis between lung functions and continuous variables. A two-sided *P* value < 0.05 was considered statistically significant. Statistical analyses were performed using SPSS version 20.0 software (IBM Corp., Armonk, NY, USA).

## Results

A total of 433 subjects participated in the study. Male athletes numbered 56.1%. Mean age was 19.82 ± 1.30 years. Body mass index (BMI) was 22.37 ± 3.51 kg/m^2^. Most subjects (41.1%) were in the first year of university education. Grade point average (GPA) was 2.86 ± 0.44. The most common sport type was the ball group (34.2%). Most subjects (74.4%) did exercise often to very often (see Table 1).

**Table 1:** Demographic and baseline characteristics of university athletes.

Characteristics	N = 433
Age, years	19.82 ± 1.30
**Gender**	
Male	243 (56.1)
Female	190 (43.9)
Body mass index, kg/m^2^	22.37 ± 3.51
Grade point average	2.86 ± 0.44
**Undergraduate education (bachelor’s degree)**	
First year	178 (41.1)
Second year	101 (23.3)
Third year	90 (20.8)
Fourth year	61 (14.1)
Fifth year	3 (0.7)
**Sport type**	
Ball group	148 (34.2)
Strength group	125 (28.9)
Static group	95 (21.9)
Endurance group	45 (10.4)
Swim group	20 (4.6)
**Exercise frequency**	
Not often (1–2 days/week)	111 (25.6)
Often (3–5 days/week)	258 (59.6)
Very often (6–7 days/week)	64 (14.8)
**Smoking**	
Age at onset of smoking, years	16.69 ± 2.43
Former smokers	60 (13.9)
Current smokers	103 (23.8)
**Tobacco characteristics**	
Amount of smoking, cigarettes per day	3.00 ± 3.20
Fagerstrom score, points	0.76 ± 1.47
Exhaled carbon monoxide, ppm	3.04 ± 2.65
Family member who smokes	121 (27.9)
Friend who smokes	120 (27.7)
Instructor who smokes	18 (4.2)
Alcohol consumption	133 (30.7)
History of asthma	24 (5.5)

Data shown as mean ± SD or n (%).

The prevalence of smoking among university athletes was 37.6% (26.5% of the athletes were males who smoke and 11.1% of the athletes were females who smoke); 13.9% were former smokers and 23.8% were current smokers. Smokers began smoking at age of 16.69 ± 2.43 years. Typical tobacco cigarettes were found to be the most common tobacco products (78.5%). Mean tobacco use was 3.00 ± 3.20 cigarettes per day. The Fagerstrom score for nicotine dependence in smokers was 0.76 ± 1.47. Asthma history was reported in 5.5% of the subjects (see Table 1).

Table 2 shows lung functions in university athletes.

**Table 2:** Lung function data of university athletes.

Variables	Pre-bronchodilator	Post-bronchodilator	*P* value
FVC, L	3.58 ± 0.75	3.68 ± 0.85	0.249
FVC, % predicted	83.80 ± 9.98	86.08 ± 14.68	0.191
FEV_1,_ L	3.07 ± 0.54	3.21 ± 0.74	0.036
FEV_1,_ %predicted	80.96 ± 9.65	86.88 ± 15.29	0.004
FEV_1_/FVC, %	82.16 ± 7.33	71.92 ± 34.07	0.066
FEF_25-75_, L/s	3.10 ± 0.67	3.82 ± 1.20	< 0.001
FEF_25-75_, %predicted	74.44 ± 22.80	88.06 ± 26.30	< 0.001

Data shown as mean ± SD.

*FVC* forced vital capacity, *FEV*_*1*_ forced expiratory volume in one second, *FEF*_*25–75*_ forced expiration flow rate at 25–75% of forced vital capacity, *L* liter, *L/s* liter/second.

When compared to non-smokers, significantly more smokers were male, and smokers tended to: be a little bit younger, have higher BMI, have lower GPA, exercise more frequently, and have higher exhaled CO levels. More smokers than non-smokers had a family member, a friend, or an instructor who smokes, and more smokers than non-smokers consumed alcohol. Furthermore, smokers had higher pre-bronchodilator FVC and FEV_1,_ and lower FEV_1_/FVC than non-smokers (see Table 3).

**Table 3:** Comparison of baseline characteristics and lung functions between smokers and non-smokers among university athletes.

Variables	Smokers n = 163	Non-smokers n = 270	*P* value
Age, years	19.98 ± 1.28	19.59 ± 1.27	0.005
Male	115 (70.6)	128 (47.4)	< 0.001
Female	48 (29.4)	142 (52.6)	< 0.001
Body mass index, kg/m^2^	22.81 ± 3.34	21.96 ± 3.53	0.021
Grade point average	2.71 ± 0.38	2.99 ± 0.45	< 0.001
Athletes who exercise often to very often	150 (92.0)	153 (56.7)	< 0.001
Exhaled carbon monoxide, ppm	3.75 ± 3.08	2.18 ± 0.73	< 0.001
Family member who smokes	64 (39.3)	48 (17.8)	< 0.001
Friend who smokes	120 (73.6)	0 (0)	< 0.001
Instructor who smokes	18 (11.0)	0 (0)	< 0.001
Alcohol consumption	133 (81.6)	0 (0)	< 0.001
History of asthma	9 (5.5)	13 (4.8)	0.754
History of allergic rhinitis	9 (5.5)	7 (2.6)	0.423
**FVC, L**			
Pre-bronchodilator	3.82 ± 0.80	3.58 ± 2.66	0.298
Post-bronchodilator	3.82 ± 0.97	3.59 ± 0.81	0.443
**FVC, % predicted**			
Pre-bronchodilator, % predicted	89.65 ± 17.61	83.22 ± 15.72	0.001
Post-bronchodilator, % predicted	89.14 ± 21.69	83.41 ± 8.77	0.359
**FEV**_**1,**_** L**			
Pre-bronchodilator	3.28 ± 0.62	2.99 ± 0.72	< 0.001
Post-bronchodilator	3.33 ± 0.83	3.13 ± 0.72	0.377
**FEV**_**1,**_** %predicted**			
Pre-bronchodilator	92.60 ± 15.36	87.77 ± 11.23	0.002
Post-bronchodilator	88.80 ± 20.36	84.31 ± 11.17	0.444
**FEV**_**1**_**/FVC, %**			
Pre-bronchodilator	78.21 ± 5.38	79.70 ± 5.60	0.015
Post-bronchodilator	60.98 ± 41.37	80.74 ± 23.89	0.066
**FEF**_**25-75**_**, L/s**			
Pre-bronchodilator	3.19 ± 0.85	3.03 ± 0.79	0.077
Post-bronchodilator	4.01 ± 1.40	3.70 ± 1.14	0.474
**FEF**_**25-75**_**, %predicted**			
Pre-bronchodilator	95.65 ± 20.13	96.08 ± 22.63	0.860
Post-bronchodilator	93.49 ± 31.38	83.98 ± 22.37	0.287

Data shown as mean ± SD or n (%).

*FVC* forced vital capacity, *FEV*_*1*_ forced expiratory volume in one second, *FEF*_*25–75*_ forced expiration flow rate at 25–75% of forced vital capacity, *L* liter, *L/s* liter per second.

## Discussion

This study is a cross-sectional research that found relatively high smoking prevalence among varsity athletes in a university in Thailand (23.8% were current smokers), similar to the study of university students by Dugral et al.^21^ (26.6% male and 15.6% female smoked). These rates were higher than those for college and university athletes in the U.S. according to studies by Gingiss et al.^1^ (4% were current cigarette smokers) and Primack et al.^2^ (16.4% were current cigarette smokers), and also higher than the rate for professional athletes in Iran as reported by a study of Hessami et al.^3^ (9% were current cigarette smokers). Smoking prevalence in our study was higher than in the general population of Thailand (19% in 2017)^22^.

This study aimed to explore characteristics of lung functions among university athletes who were smokers compared to non-smokers, therefore a subject who was unable to perform spirometry or exhaled CO test would be excluded. However, all subjects in our study could perform spirometry and exhaled CO test.

Our study showed that smokers had significantly better pre-bronchodilator FVC and FEV_1_, but lower FEV_1_/FVC ratios than non-smokers. Higher lung function values in smokers may result from more frequent exercise than non-smokers according to our data. Similarly, a prospective cross-sectional study of university students by Dugral E, et al. found that smoking could preserve lung functions in these young adults; they were called healthy smokers^21^. These findings were reviewed and described by Becklake et al.^23^ which found good lung function in smokers in several studies. No scientific plausibility could explain a mechanism in these phenomena^23^. Although smokers did not show chronic symptoms or abnormal lung functions, they did show subtle changes in lung morphology, lung inflammation and lung function^9^. However, smoking was associated with an increased rate of function decline over time^9^. A study of passive smoking in young athletes by Goic-Barisic et al.^14^ found that those who were passively exposed to smoking had worse FVC and FEV_1_ than those who were not exposed to tobacco smoke. Our study showed that presence of lower FEV_1_/FVC ratio in smokers may indicate a trend towards increased risk of airway obstruction in the future although the ratio was not less than 0.70 applied in the diagnosis criteria of COPD^24^. If these young smokers can stop smoking as soon as possible, this strategy would be a protective factor against developing several diseases, especially COPD and lung cancer. Smoking cessation clearly improved respiratory symptoms and bronchial hyperresponsiveness, and prevented excessive decline in lung function^9^.

Risk factors for cigarette smoking in our study were family member smoking, friend smoking, instructor smoking, and alcohol consumption. These social factors excluding family member smoking may be corrected by institute policies and procedures such as smoke-free university policies which provide smoking zones in very remote outdoor areas, smoking cessation clinics, social networks within the institutes, and campaigns and media against tobacco. These policies have been adopted for our university for at least 10 years. Review of literature by Hahn^25^ showed that smoke-free legislation has health and economic impacts and that the outcomes may have more to do with implementation effectiveness than adoption. Interestingly, our results showed all non-smokers consumed no alcohol. This finding suggests that sobriety is a usual social behavior at Thammasat University. This differs from the behavior of smokers in this study who often consumed alcohol as well as tobacco.

Several factors can induce a person to drink or smoke during the initial phase of alcohol or tobacco use. Most drinkers develop a pattern of social drinking; only a few drinkers become dependent on alcohol. Conversely, the majority of smokers become nicotine dependent; only a minority of smokers continue a pattern of social use^26^.

This study has a few limitations. Firstly, this study did not collect data on respiratory symptoms and signs of asthma or allergic rhinitis which subjects may have underestimated for diagnosis, but these data were not the main objective of the study. Secondly, smokers might have quit smoking in preparation for exhaled CO and spirometry testing, resulting in false-low exhaled CO levels and better lung function parameters. Lastly, we cannot predict lung function changes in the future because this study is a cross-sectional study by nature. Therefore, a prospective study may be needed to determine changes in lung functions among smokers and non-smokers among university athletes.

## Conclusions

Prevalence of smoking among university athletes was relatively high, although nicotine addiction was very low and lung functions were still good. Home and institute environments had important influences on cigarette use in students. Some strategies are needed to develop effective prevention and intervention approaches.

## References

[1] Gingiss PL, Gottlieb NH (1991). A comparison of smokeless tobacco and smoking practices of university varsity and intramural baseball players. Addict Behav..

[2] Primack BA, Fertman CI, Rice KR, Adachi-Mejia AM, Fine MJ (2010). Waterpipe and cigarette smoking among college athletes in the United States. J. Adolesc. Health.

[3] Hessami Z, Aryanpur M, Emami H, Masjedi M (2012). Behavior and knowledge of Iranian professional athletes towards smoking. Asian J. Sports Med..

[4] Alaranta A (2006). Snuff use and smoking in Finnish olympic athletes. Int. J. Sports Med..

[5] Perez-Rios M (2009). Fagerstrom test for nicotine dependence vs heavy smoking index in a general population survey. BMC Public Health.

[6] Deveci SE, Deveci F, Acik Y, Ozan AT (2004). The measurement of exhaled carbon monoxide in healthy smokers and non-smokers. Respir. Med..

[7] Chatrchaiwiwatana S, Ratanasiri A (2017). Exhaled carbon monoxide levels among tobacco smokers by age. Southeast Asian J. Trop. Med. Public Health.

[8] Ezzati M, Henley SJ, Thun MJ, Lopez AD (2005). Role of smoking in global and regional cardiovascular mortality. Circulation.

[9] Willemse BW, Postma DS, Timens W, ten Hacken NH (2004). The impact of smoking cessation on respiratory symptoms, lung function, airway hyperresponsiveness and inflammation. Eur. Respir. J..

[10] Vineis P (2004). Tobacco and cancer: recent epidemiological evidence. J. Natl. Cancer Inst..

[11] Jang DJ, Kim HC, Kim JK, Jung SY, Kim DY (2017). Effects of habitual smoking on cardiopulmonary function in taekwondo athletes. J. Exerc. Rehabil..

[12] Packa-Tchissambou B (2001). Effect of smoking on weight and cardiopulmonary capacities in young athletes. Sante.

[13] Tsimoyianis GV (1987). Reduction in pulmonary function and increased frequency of cough associated with passive smoking in teenage athletes. Pediatrics.

[14] Goic-Barisic I (2006). Influence of passive smoking on basic anthropometric characteristics and respiratory function in young athletes. Coll. Antropol..

[15] Nasser AMA, Geng Y, Al-Wesabi SA (2020). The prevalence of smoking (cigarette and waterpipe) among university students in some Arab countries: a systematic review. Asian Pac. J. Cancer Prev..

[16] Ryan H, Trosclair A, Gfroerer J (2012). Adult current smoking: differences in definitions and prevalence estimates–NHIS and NSDUH, 2008. J. Environ. Public Health..

[17] Goldstein AO, Gans SP, Ripley-Moffitt C, Kotsen C, Bars M (2018). Use of expired air carbon monoxide testing in clinical tobacco treatment settings. Chest.

[18] Miller MR (2005). General considerations for lung function testing. Eur. Respir. J..

[19] Miller MR (2005). Standardisation of spirometry. Eur. Respir. J..

[20] Heatherton TF, Kozlowski LT, Frecker RC, Fagerstrom KO (1991). The Fagerstrom test for nicotine dependence: a revision of the Fagerstrom tolerance questionnaire. Br. J. Addict..

[21] Dugral E, Balkanci D (2019). Effects of smoking and physical exercise on respiratory function test results in students of university: a cross-sectional study. Medicine (Baltimore).

[22] Aungkulanon S (2019). Smoking prevalence and attributable deaths in Thailand: predicting outcomes of different tobacco control interventions. BMC Public Health.

[23] Becklake MR, Lalloo U (1990). The 'healthy smoker': a phenomenon of health selection?. Respiration.

[24] Vogelmeier CF (2017). Global strategy for the diagnosis, management, and prevention of chronic obstructive lung disease 2017 report. GOLD executive summary. Am. J. Respir. Crit. Care Med..

[25] Hahn EJ (2010). Smokefree legislation: a review of health and economic outcomes research. Am. J. Prev. Med..

[26] Little HJ (2000). Behavioral mechanisms underlying the link between smoking and drinking. Alcohol Res. Health..

